# Kidney transcriptome and cystic kidney disease genes in zebrafish

**DOI:** 10.3389/fphys.2023.1184025

**Published:** 2023-05-04

**Authors:** Matthew Koslow, Ping Zhu, Chantal McCabe, Xiaolei Xu, Xueying Lin

**Affiliations:** ^1^ Department of Biochemistry and Molecular Biology, Mayo Clinic, Rochester, MN, United States; ^2^ Department of Biomedical Statistics and Informatics, Mayo Clinic, Rochester, MN, United States; ^3^ Department of Cardiovascular Medicine, Mayo Clinic, Rochester, MN, United States

**Keywords:** zebrafish, cystic kidney disease, sexual diamorphism, transcriptome, ciliopathy

## Abstract

**Introduction:** Polycystic kidney disease (PKD) is a condition where fluid filled cysts form on the kidney which leads to overall renal failure. Zebrafish has been recently adapted to study polycystic kidney disease, because of its powerful embryology and genetics. However, there are concerns on the conservation of this lower vertebrate in modeling polycystic kidney disease.

**Methods:** Here, we aim to assess the molecular conservation of zebrafish by searching homologues polycystic kidney disease genes and carrying transcriptome studies in this animal.

**Results and Discussion:** We found that out of 82 human cystic kidney disease genes, 81 have corresponding zebrafish homologs. While 75 of the genes have a single homologue, only 6 of these genes have two homologs. Comparison of the expression level of the transcripts enabled us to identify one homolog over the other homolog with >70% predominance, which would be prioritized for future experimental studies. Prompted by sexual dimorphism in human and rodent kidneys, we studied transcriptome between different sexes and noted significant differences in male vs. female zebrafish, indicating that sex dimorphism also occurs in zebrafish. Comparison between zebrafish and mouse identified 10% shared genes and 38% shared signaling pathways. String analysis revealed a cluster of genes differentially expressed in male vs. female zebrafish kidneys. In summary, this report demonstrated remarkable molecular conservation, supporting zebrafish as a useful animal model for cystic kidney disease.

## 1 Introduction

Polycystic kidney disease (PKD) is a condition where fluid filled cysts form on kidneys. PKDs can be separated into two distinct groups, either Autosomal Dominant PKD (ADPKD) or Autosomal Recessive PKD (ARPKD) (Harris and Torres, 2009). ADPKD is the common form of PKDs and typically affects one out of 1,000 people across various genetic backgrounds with a reported 10 million people affected globally (Bergmann et al., 2018). ADPKD patients have microcysts since childhood and massively enlarged cysts and kidney function decline around the age of 50, many of them eventually develop renal failure and require dialysis or a kidney transplant. By contrast, ARPKD is a rarer and more severe disease than ADPKD (Torres and Harris, 2014; Bergmann et al., 2018). Because proteins encoded by PKD associated genes localize in the primary cilium, in addition to other cellular places, or affect cilia-mediated signaling, PKD is largely considered as a ciliopathy (Gerdes et al., 2009; Hildebrandt et al., 2011; Hu and Harris, 2020). Renal cysts are also commonly presented in other types of ciliopathies, such as Bardet-Biedl Syndrome, Senior Loken Syndrome, Joubert Syndrome, Meckel-Gruber Syndrome, and Nephronophthisis, which are collectively referred as syndromic forms of PKD (Fliegauf et al., 2007; McConnachie et al., 2021). At this time, tolvaptan, a vasopressin V2 receptor antagonist that lowers the intracellular cAMP level, is the only FDA-approved drug to slow ADPKD progression (Chebib et al., 2018; Zhou and Torres, 2023). Thus, it is imperative to investigate potential therapies going forward.

While rodent models have been predominantly used to study PKD, there have been recent advances in utilizing zebrafish (*Danio rerio*) to study PKD as well. Zebrafish belongs to the teleosts that account for nearly half of the extant vertebrate species (Volff, 2005). The zebrafish pronephric kidney is comprised of a pair of tubules that are fused at a glomerulus. It has conserved nephrotic structure and morphogenesis processes comparable to the mammalian metanephric kidney (Drummond et al., 1998; Wingert et al., 2007). Zebrafish has become an attractive model for studying various ciliopathies because it provides a convenient *in vivo* platform for analyzing the structure, number, and length of cilia (Song et al., 2016). Additionally, zebrafish develop cysts when genes associated with human PKDs are mutated such as *pkd1*, *pkd2* and *hnf1b* (Sun and Hopkins, 2001; Sun et al., 2004; Sullivan-Brown et al., 2008; Mangos et al., 2010; Zhu et al., 2017). The adult zebrafish mesonephric kidney has a largely conserved branching morphogenesis and segment composition as mammals (McCampbell et al., 2014; Diep et al., 2015). Adult zebrafish mutants of *tmem67,* whose human homolog *TMEM67* is a major causative gene for Meckel-Gruber Syndrome (Smith et al., 2006; Leightner et al., 2013), have been shown to develop progressive renal cysts (Zhu et al., 2021).

Despite these attributes of the zebrafish model to cystic kidney diseases, there are several concerns on the conservation of the fish model. First, while mammals have primary cilia in the kidney, cilia in the zebrafish kidney are motile and cilia motility is closely associated with pronephric cyst formation (Kramer-Zucker et al., 2005; Becker-Heck et al., 2011). Second, unlike mammalian kidneys that are metanephros, adult zebrafish kidneys are mesonephros (Davidson, 2011). Third, it has been estimated that approximately 70% genes overlap between humans and zebrafish, however, the percentage of human PKD associated genes with zebrafish homologs is unknown (Howe et al., 2013). Fourth, given that approximately 30% zebrafish genome is duplicated early in the evolution of teleost fish, >100 million years ago, genetic analysis of genes with multiple homologs could be complicated (Woods et al., 2000; Howe et al., 2013). While zebrafish has proven to be an efficient model for genetic studies of disease causative genes, particularly useful for the identification of genetic modifiers for diseases such as cardiomyopathy (Ding et al., 2011; Ding et al., 2016; Ma et al., 2020), these concerns have hindered using zebrafish for genetic studies of PKD. Thus, there is an urgent need of assessing whether zebrafish is a conserved animal model for PKD research.

In the kidney, sexual dimorphism in gene expression patterns, transporters, responses to injury, as well as PKD progression has been reported (Stewart, 1994; Menezes et al., 2016; Veiras et al., 2017; Laouari et al., 2022; Neugarten and Golestaneh, 2022). In humans, male kidneys are usually larger than female kidneys, suggesting differing developmental dynamics (Coggins et al., 1998; Cheong et al., 2007; Kalucki et al., 2020). Renal function decreases with age, however, older male patients with chronic kidney disease usually are more prone to kidney failure, particularly as there are disruptions in the nitric oxide system (Eriksen and Ingebretsen, 2006; Baylis, 2009). Comparing with age-matched female patients, male patients with ADPKD consistently manifest more severe phenotypes, including lower estimated glomerular filtration rate and larger height-adjusted total kidney volume, and earlier onset of End-Stage Renal Disease (Neugarten et al., 2000; Reed et al., 2008). Similar to humans, sexual dimorphism has been also reported in animal models, including Han:SPRD rats and *Pkd1* mutant mice (Nagao et al., 2005; Stringer et al., 2005; Menezes et al., 2016). Consistent with the phenotypic changes, differences in total RNA transcripts (transcriptome) have been reported in genes and pathways between male and female mouse kidneys (Menezes et al., 2016; Terabayashi et al., 2020). In zebrafish, the observation of renal cysts mainly in male *tmem67* mutants suggests sexual dimorphism in the kidney, however, whether male and female fish have distinct transcriptome remains unknown (Zhu et al., 2021).

In this manuscript, we decided to take two approaches to assess conservation of zebrafish for PKD study. First, we assessed molecular conservation by querying how many cystic kidney disease genes have their corresponding zebrafish homologs. We found that out of 82 genes that cause renal cysts, zebrafish have homologs for 81 of these genes. Second, we compared gene expression between male and female fish kidneys and noted obvious differences. Among the differentially expressed signaling pathways, we noted a remarkable 37.9% similarities to a previously established rodent PKD model (Menezes et al., 2016). Together, our studies indicate zebrafish a valuable model for studying PKD.

## 2 Materials and methods

Experimental design was summarized in a flowchart (Supplementary Figure S1).

### 2.1 Zebrafish husbandry and maintenance

Zebrafish were maintained under a 14-h light and 10-h dark cycle at 28.5°C as previously described (Shih et al., 2015). Adult WIK fish were obtained by allowing the embryos that were derived from in-house breeding to grow to 12 months of age and used in accordance with the policies of the Mayo Clinic Institutional Animal Care and Use Committee.

### 2.2 Identifying fish homologs, and calculating percent identity and relative abundance

PKD associated genes were selected from the review by McConnachie et al. (2021). Protein sequences were found on ZFIN (The Zebrafish Information Network) ( https://www.zfin.org ). In most cases, the ENSEMBL (European Bioinformatics Institute and the Wellcome Trust Sanger Institute) gene ID was provided. When ENSEMBL did not have records of this gene, we gave the ZFIN gene ID. To calculate the percent identity of a gene, we searched https://www.uniprot.org/, aligned the Uniprot IDs of the zebrafish and human genes, and calculated the percent identity. To compare relative abundance of genes with multiple homologs, we added the Reads Per Kilobase of transcript per Million reads mapped (RPKM, a measure of relative RNA molar concentration of a transcript in a sample) values of each homolog together for the total abundance as previously described (Shih et al., 2015). We then divided the single value RPKM of a specific homolog to the total RPKM of the multiple homologs in order to get a percentage.

### 2.3 RNA isolation

Kidneys of 12-month-old male and female fish were isolated as previously described (Ding et al., 2022). Briefly, zebrafish were anesthetized via tricaine, and kidneys were harvested as needed. A total of 10 sets of kidneys were harvested, including 5 sets of male and female kidneys, respectively. The kidneys were submerged in TRIZOL (Sigma) and metal beads were added to help dissociate the tissue. After dissociation in TRIZOL, chloroform was added to separate out the aqueous layer. This aqueous layer was then precipitated in isopropanol to isolate the RNA, before reconstituted in RNAase free water. RNA samples were considered good quality with an RNA Integrity Number (RIN) greater than seven.

### 2.4 RNA sequencing and analysis

RNA libraries were generated, and RNA sequencing was conducted at the Mayo Clinic Medical Genome Facility, a core laboratory offering research sequencing. The paired-end RNA reads were processed through RapMap, a custom pipeline developed by the Mayo Clinic Bioinformatics core sequencing analyses of species with small genomes. RapMap utilizes Kallisto (Bray et al., 2016) for pseudoalignment and transcript quantifications, with bootstrapping enabled to *n* = 100 (Bray et al., 2016). Bootstrapping is enabled while running Kallisto quantification to allow for quantifying transcript abundance. The most recently available zebrafish genome, version GRCz11, Ensembl gtf version GRCz11.101 was used for alignment. In total, 222,675 genes were identified mapping to this reference genome. RapMap also utilizes Tximport (Srivastava et al., 2016) for summarizing transcript abundances for downstream analysis and MultiQC (Ewels et al., 2016) for overall quality control. Samples with over 75% of reads mapping to the genome were considered high quality. Raw and processed files were submitted to the NCBI GEO under accession number GSE2248592. Additionally, mouse RNA-seq data was downloaded from a previous study (Geo Omnibus number GSE72554) (Menezes et al., 2016).

### 2.5 Identifying and characterizing differentially expressed genes

Using the resulting length ScaledTPM (TPM scaled up to library size) values, differentially expressed (DE) genes (FDR <0.05, log2fold change > |1.5|) were assessed using the bioinformatic package edgeR (Robinson et al., 2010). Canonical pathway analysis was performed with the DE genes using Ingenuity pathway analysis (IPA) software (QIAGEN) (Kramer et al., 2014). The top 10 canonical pathways in each species were identified and compared. Individual genes within each pathway were examined further. Additionally, we uploaded our DE genes into the STRING database, a database of known and predicted protein-protein interactions (string-db.org) (Szklarczyk et al., 2021), to identify relationships among our DE genes. The STRING network was generated and visualized at the highest confidence (0.9) level and disconnected nodes or clusters with fewer than 3 genes were removed. IPA analysis was performed on genes in the resulting network.

### 2.6 qPCR

We picked the top 10 most differentially expressed genes that were found via STRING network analysis, which included the top 5 upregulated and top 5 downregulated genes as indicated in section 2.5. The Superscript III First-Strand Synthesis System (Invitrogen) was used to generate cDNA from 500 ng of RNA that was isolated in section 2.4. Once cDNA had been successfully generated, quantitative PCR (qPCR) was carried out using QuantStudio 7 Pro (Thermo Fischer) QPCR apparatus in 96-well qPCR plates (Roche Diagnostics Corp). The expression of the genes was normalized using the expression level of the housekeeping gene *gapdh* and its corresponding –∆∆Ct (cycle threshold) values. Nine RNA samples were analyzed in triplicate. Primer sequences for each gene can be found in Supplementary Table S3. Statistical analysis was conducted via vassarstats (http://vassarstats.net/). Statistically significant DEGs between male and female zebrafish were indicated by two tailed *t-test* with a minimum significance of *p* < 0.05.

## 3 Results

### 3.1 99% cystic kidney disease-associated genes have corresponding zebrafish homologs

**FIGURE 1:**
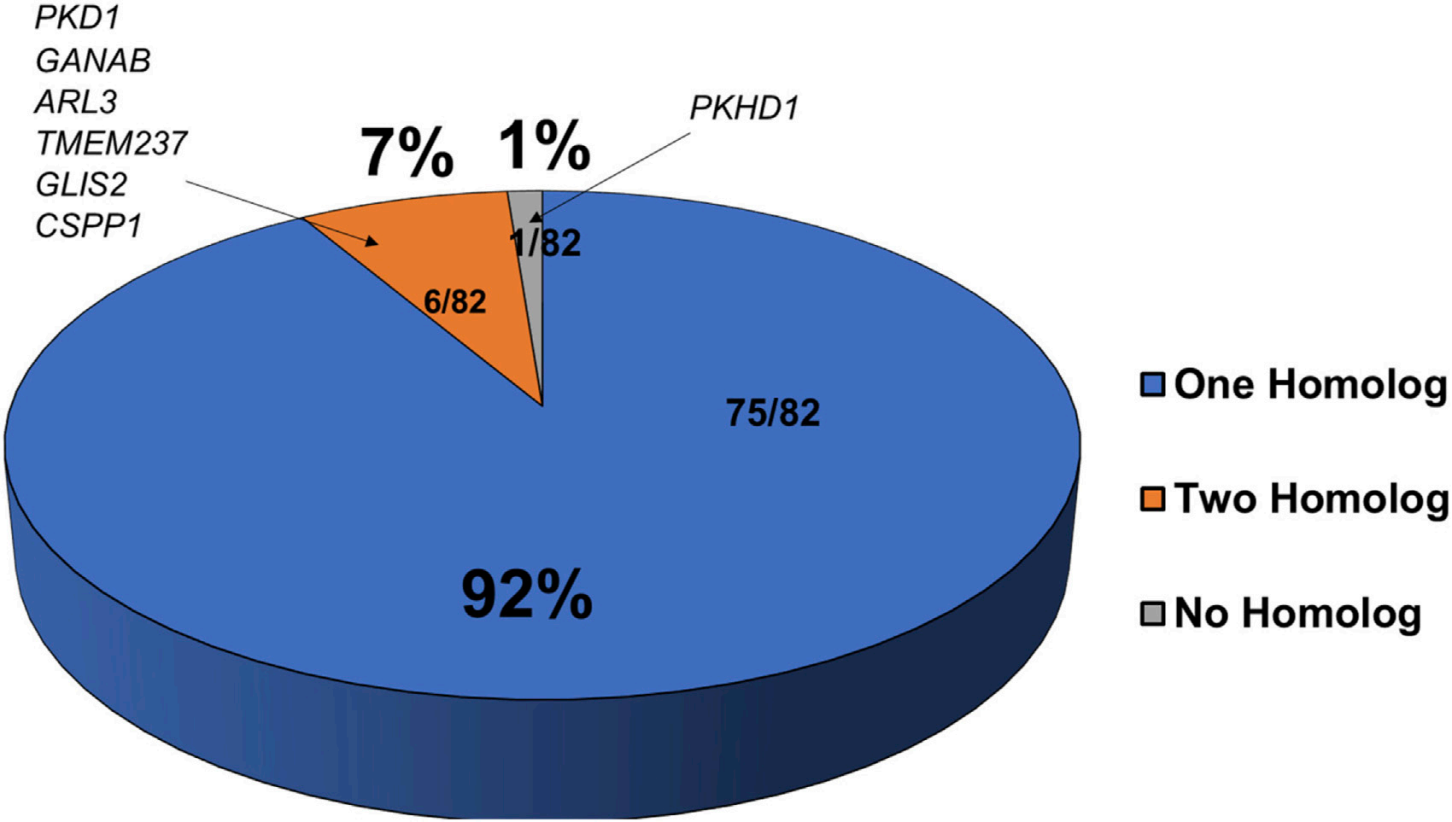
Majority of cystic kidney disease associated genes have corresponding homologs in zebrafish. Shown is a pie chart representing the number of zebrafish homologs exist for known human PKD associated genes. Genes with one zebrafish homolog are in blue, those with two homologs are in orange, and those with no known homologs are in grey.

To test molecular conservation of the zebrafish, we queried the zebrafish genome to identify homologs of known cystic kidney disease related genes. Based on inherited pattern and phenotypes in patients, we decided on 82 genes that were separated into multiple categories including ADPKD, ARPKD, and disorders with significant presence of kidney cysts (Reiter and Leroux, 2017; McConnachie et al., 2021). Many of these gene encode proteins in renal cilia, underscoring the importance of cilia in PKD pathogenesis. We compared gene orthologs of these human genes to the ZFIN database (http://zfin.org) and Ensembl (http://www.ensembl.org/index.html) to determine if zebrafish homologs exist for these genes (Supplementary Table S1). We were able to identify zebrafish homologues for 81 of the 82 genes, indicating a 99% overlap of expression across species (Figure 1). At the protein level, the identities of each of these genes relative to their human counterparts ranged from 30% to 95% with an average of around 59% (Supplementary Table S1). All ADPKD-associated genes, including major causative gene *PKD1* and *PKD2* as well as genes recently linked to ADPKD such as *IFT140*, *DNAJB11*, *GANAB*, and *ALG9*, have zebrafish homologs (Supplementary Table S1) (Porath et al., 2016; Cornec-Le Gall et al., 2018; Besse et al., 2019; Senum et al., 2022). However, we were unable to find a zebrafish homolog of ARPKD causative gene *PKHD1*, even after searching the National Center for Biotechnology Information database using BLASTX (Figure 1 and Supplementary Table S1) (Ward et al., 2002). The closest existing homolog in zebrafish is *pkhd1l* (*PKHD1* like), with an identity of 52% relative to its direct human homolog. Moreover, 75 of these 81 genes have a single homolog, while only 6 of these 81 genes have two separate homologs. These genes were *pkd1, ganab, arl3, tmem237, glis2,* and *cpps1* (Figure 1 and Supplementary Table S1)*.*

### 3.2 Transcriptome studies in kidney aid the prioritization of zebrafish kidney cyst-associated genes for future studies

We then defined gene expression of 12-month-old zebrafish kidney via carrying RNA sequencing (RNAseq) analysis. Both male and female fish samples were extracted with 5 sets of kidneys of each sex. We used an RPKM value of 0.3 as our cutoff for expression abundance. The average number of total reads per sample was 45,149,820 with an average of 35,128,575 reads mapped to a gene. We were able to map an average of 77% of these reads to the reference genome, enabling us to analyze expressional changes of 22,676 genes. Based on their expression abundance in zebrafish kidney transcriptomes, 81 cystic kidney disease-associated genes can be broadly divided into four categories, high, medium, low, and undetectable (Supplementary Table S2), referred as Group 1, Group II, Group III, and Group IV, respectively. Genes that comprised of Group I (with RPKM values greater than 1,000) were *ganaba*, *ganabb*, *dnajb11*, *armc9*, and *ift74* in both male and female kidneys, *poc1b* and *xpnpep3* in male kidneys, and *cep290* in female kidneys (Supplementary Table S2). Genes that comprised of group II was the largest category of genes (with RPKM values between 100 and 999). Genes in group III (with RPKM values between 1 and 99) included many BBS adjacent genes (*bbs1, bbs4, bbs5, bbs9, bbs10,* and *ttc8*). The expression of the following genes in group IV had their RPKM values below threshold: *c8orf37*, *ift74*, *cep164*, *cplane1*, *cep120*, *katnip*, *anks6*, and *cep83* (Supplementary Table S2). Although these genes had extremely low to zero expression levels in 12 months zebrafish kidney, this data cannot be interpreted as excluding these genes as kidney-expression genes. For example, it is possible that these genes are activated in the kidney during development. Indeed, *cep83* is expressed in the pronephric kidney (Joo et al., 2013); *ift74, cep164*, *cep120*, and *anks6* might also be expressed in the embryonic kidney since knockdown of these genes results in pronephric cysts (Chaki et al., 2012; Hoff et al., 2013; Shaheen et al., 2015; Lindstrand et al., 2016).

In our previous bioinformatic analysis of dilated cardiomyopathy genes in zebrafish, we demonstrated that comparison of expression levels of multiple zebrafish homologues can help to prioritize one homologue over the other (Shih et al., 2015). It is hypothesized that the greater the abundance, the more important this homolog is for function. To quantify the relative abundance, we took the raw RPKM value for each individual homologue and divided that by the total sum of both RPKM values to get a percentage. For the 6 genes with 2 homologs, we recommended that one homolog, based on the percentage, would be prioritized for future studies (Table 1). For example, *pkd1a* is more prevalent than *pkd1b* with *pkd1a* having over 90% abundance. This is consistent with our recent study showing that *pkd1a* is the major homolog of *PKD1* in zebrafish while *pkd1b* plays a redundant role (Zhu et al., 2017). We also identified the more predominant homolog for the other remaining genes: *ganabb*, *arl3b*, *tmem237b*, *glis2a*, and *cpps1b* as compared to their other homolog (Table 1).

**TABLE 1:** Predominant homolog of zebrafish genes with multiple homologs can be predicted based on their relative abundance in kidney.

Human Gene	Zebrafish Homolog	Identity (%)	Abundance (Female, RPKM)	Relative Abundance (%, Female)	Abundance (Male, RPKM)	Relative Abundance (%, Male)	Predominant homolog
***PKD1***	*pkd1a*	39.6	61.99	92.2	47.19	93.6	***pkd1a***
	*pkd1b*	30.1	5.34	7.9	3.29	6.4	
***GANAB***	*ganaba*	64.1	1035.1	24.8	1300.4	30	***ganabb***
	*ganabb*	66	3146.28	75.2	3039.12	70	
***ARL3***	*arl3a*	95.1	57.81	15.2	45.44	11.6	***arl3b***
	*arl3b*	94.5	322.28	84.8	344.94	88.4	
***TMEM237***	*tmem237a*	52.6	10.70	4	0	0	***tmem237b***
	*tmem237b*	52.3	255.91	96	137.77	100	
***GLIS2***	*glis2a*	50.5	399.28	94.8	91.16	88.2	***glis2a***
	*glis2b*	52.6	21.85	5.2	12.197	11.2	
***CSPP1***	*cspp1a*	38.6	158.88	24.3	181.33	31.4	***cspp1b***
	*cspp1b*	32.2	495.48	75.7	395.43	68.8	

### 3.3 Transcriptomic sexual dimorphism occurs in zebrafish kidneys

**FIGURE 2:**
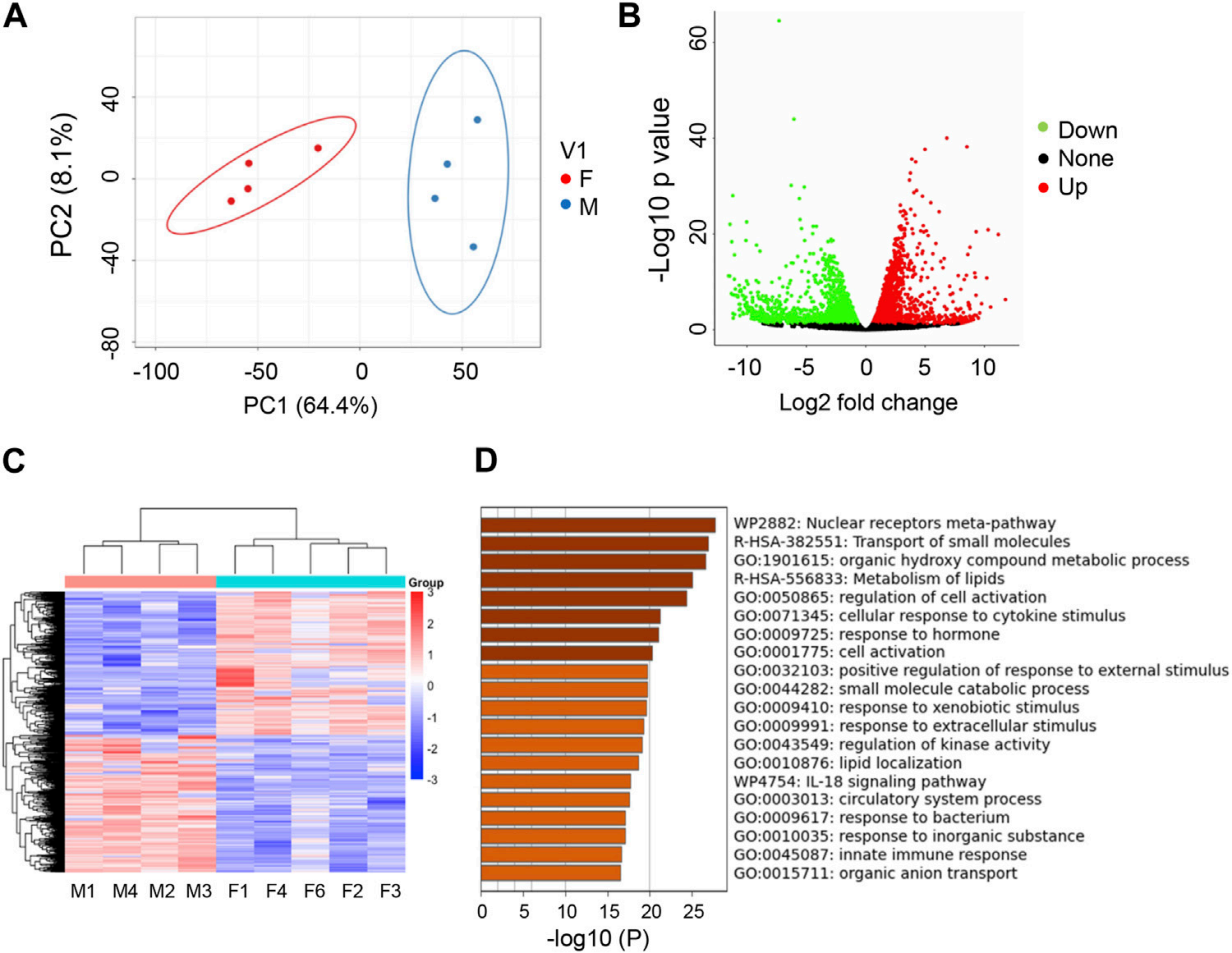
Transcriptome studies uncover sexual dimorphism in zebrafish kidneys. **(A)** PCA analysis of RNAseq data for male (M) vs. female (F) kidneys (4 replicates per group). **(B)** Volcano Plot of M vs. F kidneys (4 replicates per group). Significantly differentially expressed genes (−1.5 < log_2_FC > 1.5, FDR <5%) are highlighted green (downregulated) or red (upregulated). **(C)** Heatmap of total genes in M v F kidneys. **(D)** Metascape analysis of top differentiated pathways.

We next wanted to determine if there was sexual dimorphism at the transcriptomic level in zebrafish kidneys. Indeed, when comparing transcriptome of zebrafish kidneys of different sex, we found that transcripts in male and female kidneys form distinct clusters, with those in male fish circled in blue and those in female fish circled in red, as indicated by PCA analysis (Figure 2A). Using EdgeR, we identified a total of 2,422 DEGs between sexes (FDR <0.05, log2fold change of > |1.5|). Genes marked as green are downregulated, genes marked as red are upregulated, and genes marked as black are not differentially regulated (Figure 2B). Unbiased clustering heatmaps show distinct expression differences in genes between male and female kidneys, i.e., many genes with lower expression levels in males (shown in blue) have higher expression levels in females (shown in red), and *vice versa* (Figure 2C). To query what pathways these DEGs are involved, we used Metascape and found that the biggest differences were in the nuclear receptors pathway and pathways of transporting small molecules (Figure 2D). Other pathways of note include cell response to cytokines, kinases, xenobiotics, and extracellular components. These results indicate that genetic differences do exist between male and female zebrafish kidneys.

**FIGURE 3:**
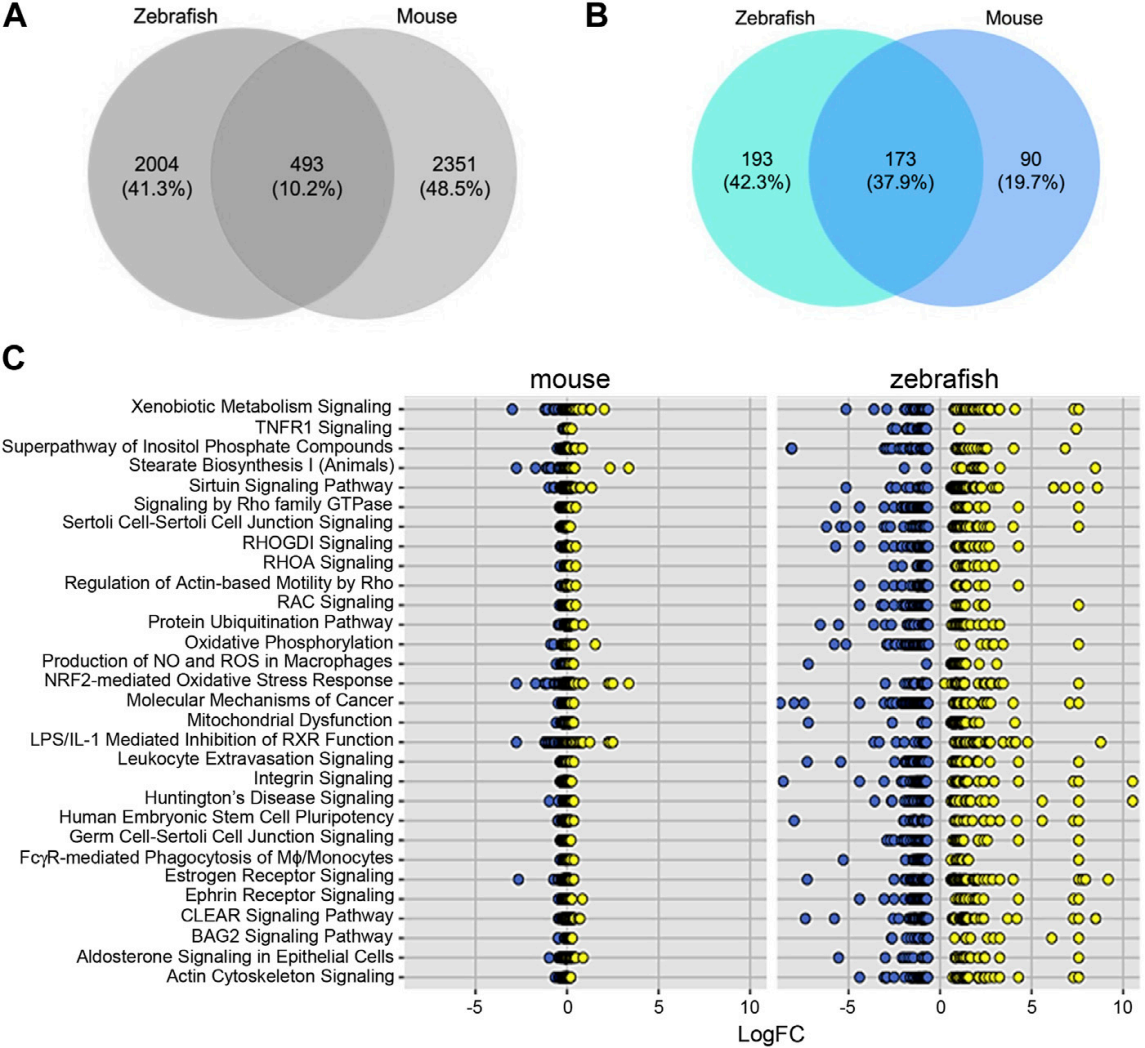
Differentially expressed genes and pathways underlying sex dimorphism are shared between zebrafish and mouse kidneys. **(A)** Venn diagram of shared genes between zebrafish and mouse. **(B)** Venn diagram of shared pathways between zebrafish and mouse. **(C)** Plot of log2fold change of shared IPA.

To further test the conservation of the zebrafish model, we sought to compare transcriptomes that define sex dimorphism in zebrafish and in mouse. In the original report, the cutoff for mouse genes was a significance of <0.05, with three to four replicates (Menezes et al., 2016). Using an FDR<5%, we identified 2,422 and 2,351 genes in zebrafish and mouse, respectively. Comparing these zebrafish and mice genes, we observed an overlap of 493 common DEGs, which is 10.2% of the total DEGs (Figure 3A). To determine if there are shared signaling pathways between zebrafish and the mouse studies, we performed ingenuity pathway analysis (IPA) on both the zebrafish and mouse genes independently. Focusing on the significant canonical pathways in each species, there were 173 shared pathways, which represent 37.9% of total significant pathways (Figure 3B). We graphed the gene expression within the top pathways to understand the expression differences of these shared pathways between species (Figure 3C; Table 2). While the direction of gene expression is similar between species, the gene expression difference between male and female was greater in zebrafish than mouse, suggesting that sexual dimorphism-associated kidney phenotypes might be more easily studied in fish than mouse. To obtain a balanced view and to determine the nature of this phenomenon, we also evaluated the different signaling pathways between zebrafish and mouse. We plotted the change in activation profile by examining the IPA canonical pathway Z-scores and comparisons were made for pathways that had an absolute Z score difference greater than 2.5 between the two species (Supplementary Figures S2A, 2B). We observed 26 pathways that fit this criterion, which include but are not limited to autophagy, PI3K-AKT signaling, and endothelin signaling amongst others. Together, these results indicate that despite some differences, molecular pathways underlying sex dimorphism are shared between zebrafish and mouse, lending credence to the zebrafish as a viable animal model to study PKD.

**TABLE 2:** Z-scores of shared pathways between sexual dimorphism in zebrafish and mouse.

Pathway	Zebrafish z-score	Mouse z-score
Actin cytoskeleton signaling	−0.544	−1.372
Aldosterone signaling in epithelial cells	−0.832	−2.041
Autophagy	0.649	0.781
BAG2 signaling pathway	2.138	−0.632
CLEAR signaling pathway	−0.911	1.612
Ephrin receptor signaling	−2.137	−0.392
Estrogen receptor signaling	0.128	−1.089
Fcg receptor-mediated phagocytosis in macrophages and monocytes	−1.768	−1.213
Human embryonic stem cell pluripotency	−0.522	−3.286
Huntington’s disease signaling	1.633	−1.134
Integrin signaling	−0.866	−2.121
Leukocyte extravasation signaling	−2.333	−2.2
LPS-stimulated MAPK signaling	−2.183	−0.447
LPS/IL-1 mediated inhibition of RXR function	−2.357	0.243
NRF2-mediated oxidative stress response	0.626	1.177
Oxidative phosphorylation	5.864	−3
RAC signaling	−2.137	−2.611
Regulation of actin-based motility by Rho	−1.3	−0.5
RHOA signaling	0.343	−1.043
RHOGDI signaling	0.801	1.095
Signaling by Rho family GTPases	−2.309	−3.683
Sirtuin signaling pathway	−1.75	1.286
Super pathway of inositol phosphate compounds	−1.581	−1.677
TNFR1 signaling	−2.524	0.905
Xenobiotic metabolism general signaling pathway	−0.577	−1.18

### 3.4 Transcriptome studies suggested candidate signaling pathways and genes for sex dimorphism in zebrafish kidney

To delve deeper on genes that is important for sex dimorphism in zebrafish, we analyzed DEGs in the following shared signaling pathway between zebrafish and mouse: Coordinated Lysosomal Expression and Regulation (CLEAR) signaling, Sirtuin and Xenobiotic Metabolism. Highlighting the CLEAR pathway, we noted 59 key genes with distinct expression patterns. Heatmaps of these 59 CLEAR genes noted clear distinctions of unique genes up or downregulated in male vs. female fish (Supplementary Figure S3A). The same could be said for the genes in the Sirtuin heatmap and the xenobiotic pathway heatmap (Supplementary Figures S3B, 3C). Genes that had the greatest fold expression for the CLEAR pathway include *rras2, tgfa, ntrk3a, tcirg1b*, and *vegefab*. Sirtuin genes with the greatest fold change included *nqo1, pck1, pfkfb3, arg2,* and *g6pd*. Xenobiotic genes that were found to have the greatest fold change include *nqo1, rras2, abcc2, il1b*, and *ugt8* (Supplementary Figure S3D). These genes in the specific pathways act as potential targets for future experimental studies to decipher underlying mechanism of sex dimorphism.

**FIGURE 4:**
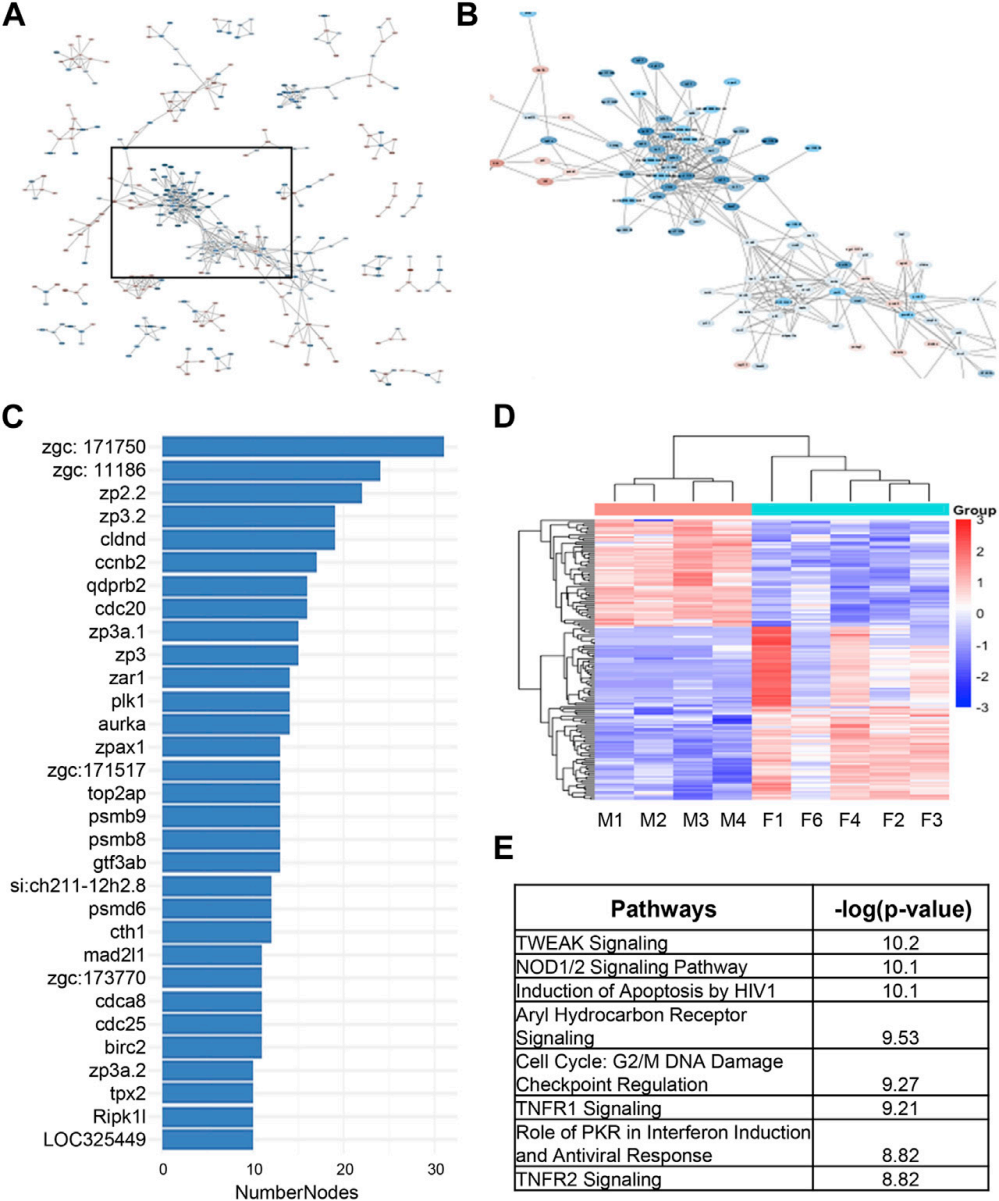
STRING analysis of differentially expressed genes underlying sex dimorphism in zebrafish kidneys. **(A)** String analysis of all DEGs in male vs. female zebrafish kidneys. Confidence score was set to 0.9. **(B)** Zoomed in area of clustered genes from (A). **(C)** Number of nodes in string cluster. **(D)** Heatmap of STRING Cluster genes in male and female zebrafish kidneys in (B). **(E)** IPA analysis of STRING Cluster genes in (B).

As an alternative way to discover candidate genes for sex dimorphism in zebrafish kidney, we used STRING analysis to identify gene clusters (Figure 4A). We were able to identify a cluster of around 150 genes closely associated to one another, many of which had large associations to one another based on the number of nodes (Figures 4B, C). The heatmap of the 150 network genes shows a clear distinction between male and female fish kidneys (Figure 4D). IPA analysis of this cluster of genes revealed enrichment of pathways involved in TNF-related weak inducer of apoptosis (TWEAK) signaling, NOD1/2 signaling, and cell cycle regulation (Figure 4E).

**FIGURE 5:**
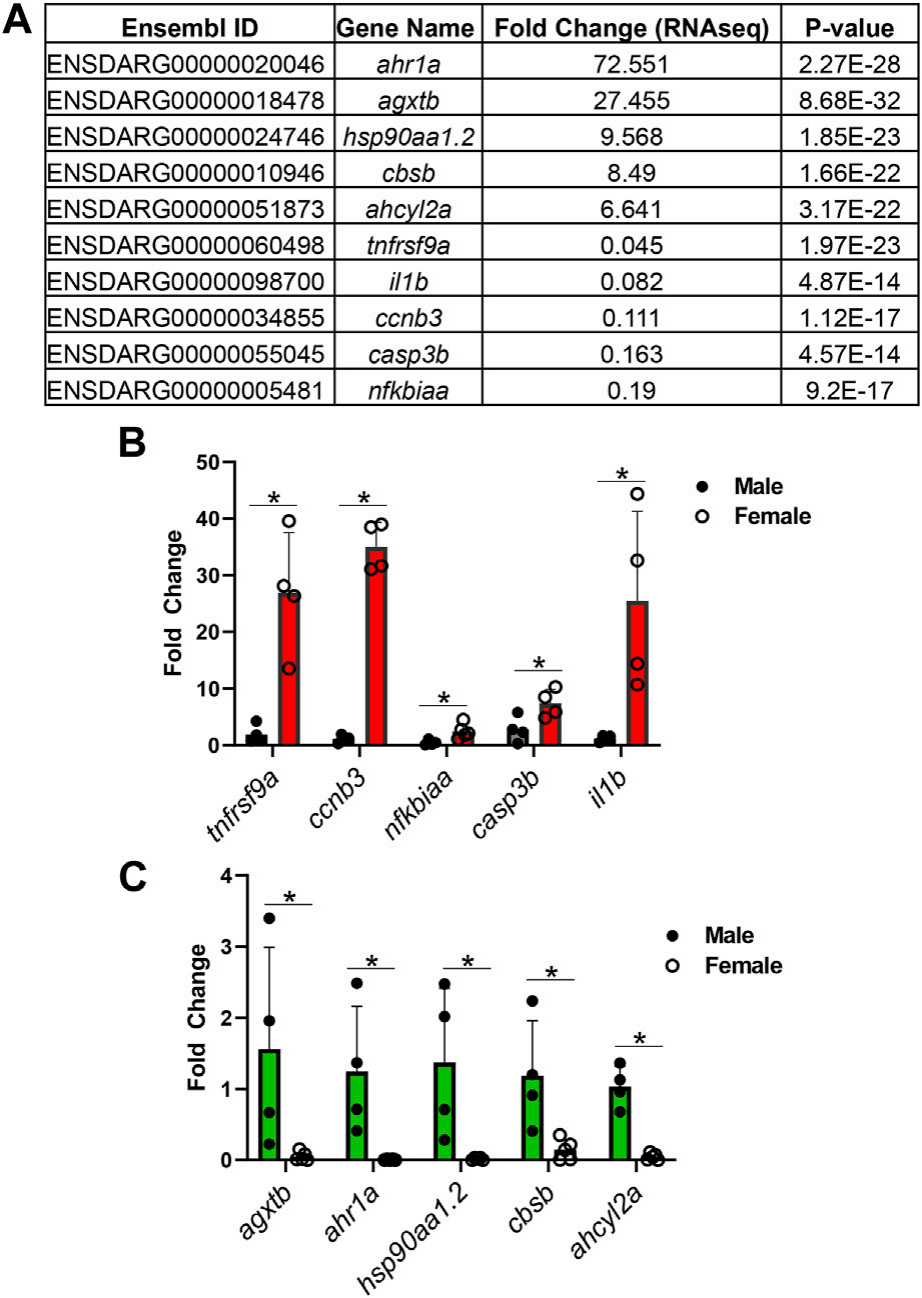
Sexually dimorphic genes from RNAseq analysis are validated by qPCR. **(A)** Table of top 10 genes and fold changes from RNAseq analysis. **(B)** qPCR validation of genes that were downregulated in male kidneys. **(C)** qPCR validation of genes that were upregulated in male kidneys. Four kidneys from male and female zebrafish were scored, respectively. *: *p* < 0.05.

Finally, to confirm the results from RNAseq analysis, we performed qPCR on the genes identified in this cluster. Consistent with the RNAseq data (Figure 5A), *ahr1a, agxtb, hsp90aa1.2, cbsb*, and *ahcyl2a* were found to be significantly upregulated in male kidney relative to female kidney (Figure 5B); *tnfrsf9a*, *nfkbiaa, casp3b*, *ccnb3,* and *ilb1* were significantly upregulated in female fish relative to male fish (Figure 5C). These results confirmed the fidelity of our RNAseq analysis for genome-wide gene expression studies.

## 4 Discussion

### 4.1 Molecular conservation of human cystic kidney disease-associated genes in zebrafish

The validity of zebrafish as a model for studying PKD in the past has come under scrutiny due to differences to mammalian models (Kramer-Zucker et al., 2005; Davidson, 2011; Howe et al., 2013). In the present manuscript, we provided the following two strong evidence to support the conservation of zebrafish for PKD studies. First, we demonstrated 99% conservation of 82 cystogenic genes, including 6 ADPKD-associated genes (*PKD1, PKD2, IFT140, DNAJB11, ALG9,* and *GANAB*), 1 ARPKD causative gene (*DZIP1L*), and 75 renal ciliopathy genes (Porath et al., 2016; Lu et al., 2017; Cornec-Le Gall et al., 2018; Besse et al., 2019; McConnachie et al., 2021; Senum et al., 2022). The only gene that did not have a zebrafish homolog was *PKHD1,* the major ARPKD gene that encodes for the Fibrocystin (Ward et al., 2002). To date there is not a known direct homolog of this gene found in zebrafish. Second, out of 81 genes, 75 have a single fish homolog, while only 6 of these genes (7.4%) have two homologs. Through abundance analysis we were able to identify which isoform is the more predominant form and should be focused upon, and thus addressed a common criticism that multiple homologs for a mammalian gene can potentially confound genetic studies in zebrafish. Our analysis indicated that renal cystic disease genes are much conserved in zebrafish than approximately 70% conservation of all genes and less expanded than the average 30% duplication (Howe et al., 2013). Importantly, our work would facilitate future experimental studies to further validate the conservation of zebrafish, via systematically generating genetic models for PKD of different etiology. When there are two or more zebrafish homologs for a certain human gene, we recommend prioritizing the predominant isoform. This notion is supported by our previous studies of *pkd1a* and *pkd1b* mutants. We showed that *pkd1a* fish develop severe phenotypes and *pkd1b* fish are generally normal (Zhu et al., 2017). For *GANAB, Tmem237*, *Cspp1*, *Arl3*, and *Gis2* that all have 2 zebrafish homologs, the predominant homolog was also predicted. It remains to be experimentally tested whether the predominant isoform would play more important roles in cyst development and progression.

### 4.2 Conservation of transcriptome underlying sexual dimorphism in zebrafish

Sexual dimorphism of kidney genes has previously been reported in mice, particularly in genes that regulate fatty acid oxidation (Menezes et al., 2016). IPA analysis on this previously published data set also revealed other key differences in signaling pathways outside of fatty acid oxidation. Here, we identified 173 total pathways that are shared between zebrafish and mice, resulting in 37% shared pathways. These pathways include xenobiotic metabolism, CLEAR signaling, and sirtuin biosynthesis, amongst others. Through comparison we also showed that there is more dynamic gene change expression in zebrafish relative to mouse. These data indicated that conservation between fish and mouse is high, justifying the zebrafish PKD model as a viable model for future studies.

One of the pathways that is affected across different model systems is the CLEAR signaling pathway. Of note, some of the genes with the greatest fold change in expression were *rras2, tgfα, ntrk3a, tcirg1b*, and *vegefα*. Of these genes, serum *VEGFα* serves as a biomarker for ADPKD progression (Reed et al., 2011; Leierer et al., 2021); *Tgfα* is upregulated in the cystic epithelia of PKD patients and transgenic overexpression of *Tgfα* promotes renal cyst formation (Gattone et al., 1996; Lee et al., 1998). TFEB is the key transcriptional regulator of genes that harbors the CLEAR motif, and TFEB has been implicated in cystic kidney disease (Sardiello et al., 2009; Calcagni et al., 2016; Shillingford and Shayman, 2023). The xenobiotic pathway is involved in the breakdown of drugs/foreign compounds. While there have not been reports establishing the link of Xenobiotic metabolism in PKD specifically, sexually dimorphic expression of xenobiotic genes has been observed in rats (Kwekel et al., 2013). Sirt1, a member of the Sirtuin pathway, has been implicated in ADPKD, as double knockouts of *Sirt1* and *Pkd1* resulted in prevention of cyst formation (Zhou et al., 2013). Future studies will be needed to demonstrate whether male vs. female zebrafish manifest different severity in modeling PKD of different etiology, as we have already noted in the *tmem67* model, in which renal cysts were mainly developed in males (Zhu et al., 2021). Several top pathways and top genes that have been uncovered here would be a great starting point to pinpoint underlying mechanism that is accountable for such sex dimorphism.

STRING/Hub analysis provided an alternative strategy to uncover a specific cluster of genes that could be important for the sex dimorphism. Some of the genes have been implicated in autophagy, ciliopathy, and kidney injury, which all play roles in PKD. For instance, *Hsp90aa1* is involved in autophagy (Macri et al., 2015). *AGXT* has been linked to ciliopathy (Gee et al., 2014). *NFKBIA* is implicated in kidney injury through DNA damage/senescence pathways (Kolesnichenko et al., 2021). Further investigation of these genes could be conducted in the future to reveal their functions in cystogenesis.

### 4.3 Implications of zebrafish as an animal model for PKD studies

Through validation of molecular conservation of PKD associated genes, the present study justified future experimental efforts to establish zebrafish as a faithful animal model for this disease. The identification of all zebrafish homologs of cystic kidney disease genes laid the foundation for systematic generation of the corresponding genetic models. Phenotyping of these future models in adult zebrafish is feasible, as exemplified by our recent study of *tmem67* mutants (Zhu et al., 2021). It is anticipated that comparison among these models could uncover both common and unique pathological events among PKD models with different etiology, which would justify the use of this lower vertebrate model for developing genotype-guided therapies. Another stringent criterion to test the fidelity of this vertebrate model would be to assess whether replacing a wild type gene with a disease-causing variant that was found in human patients (knock in) can recapitulate PKD-like phenotypes in zebrafish. If established, zebrafish could offer an efficient *in vivo* platform for evaluating large number of *PKD1* or *PKD2* variants that are discovered clinically with unknown significance.

The major driving force for establishing cystic kidney disease in a simpler but more efficient zebrafish model is the potential to conduct genetic screens. As exemplified by our recent studies in cardiomyopathy, insertional mutagenesis-based forward genetic screens can be carried out in zebrafish for systematic identification of genetic modifiers, which could lead to the identification of novel therapeutic targets such as *dnajb6* and *rxra* (Ding et al., 2011; Ding et al., 2016; Ma et al., 2020). F0-based genetic assays, which do not involve the necessity of generating stable mutants, have also been recently implemented in zebrafish for rapid assessment of modifying effects of candidate genes, such as those suggested from transcriptome studies (Ata et al., 2018; Ding et al., 2022). Both the forward genetic screen and F0-based screen could be used to search genetic modifiers of PKD. Therefore, zebrafish models have the potential to accelerate the discovery of underlying mechanisms of PKD, as well as the development of mechanism-based therapies.

## Data Availability

The datasets presented in this study can be found in online repositories. The names of the repository/repositories and accession number(s) can be found in the article/Supplementary Material.
