# Supplementary material for: Kidney transcriptome and cystic kidney disease genes in zebrafish
Source: Front Physiol. 2023 May 4;14:1184025. doi: 10.3389/fphys.2023.1184025 (PMC10226271; doi:10.3389/fphys.2023.1184025)
Supplement: Supplementary file 1 [file DataSheet1.docx]

Supplementary Table 1: Human cystic kidney disease genes with associated zebrafish homologs

| **Human cystic kidney disease associated genes** | **Gene name** | **Zebrafish homolog** | **(Ensembl) Gene ID** | **Identity (%)** | **Human Accession #** | **Kidney RPKM (F)** | **Kidney RPKM (M)** |
| --- | --- | --- | --- | --- | --- | --- | --- |
| **ADPKD** |  |  |  |  |  |  |  |
| *PKD1* | Polycystin 1 | *pkd1a pkd1b* | ENSDARG00000030417 ENSDARG00000033029 | 39.6  30.1 | P98161 | 61.99 5.34 | 47.19 3.29 |
| *PKD2* | Polycystin 2 | *pkd2* | ENSDARG00000014098 | 63.2 | Q13563 | 71.28 | 112.29 |
| *IFT140* | Intraflagellar transport 140 homolog | *ift140* | ENSDARG00000031886 | 58.5 | Q96RY7 | 393.42 | 189.76 |
| *GANAB* | Glucosidase II Alpha Subunit | *ganaba ganabb* | ENSDARG00000076811 | 64.1 66 | Q14697 | 1035.1 3146.28 | 1300.4 3039.12 |
| *DNAJB11* | DnaJ homolog subfamily B member 11 | *dnajb11* | ENSDARG00000015088 | 85 | Q9UBS4 | 2266.67 | 1772.92 |
| *ALG9* | Alpha-1,2-mannosyltransferase | *alg9* | ENSDARG00000012840 | 72.8 | Q9H6U8 | 513.43 | 376.21 |
| **ARPKD** |  |  |  |  |  |  |  |
| *PKHD1* | Fibrocystin | N/A | N/A | N/A | N/A | N/A | N/A |
| *DZIP1L* | DAZ interacting zinc finger protein 1-like | *dzip1l* | ENSDARG00000074265 | 37.1 | Q8IYY4 | 158.85 | 325.15 |
| **BBS** |  |  |  |  |  |  |  |
| *BBS1* | Bardet-Biedl syndrome 1 | *bbs1* | ENSDARG00000075169 | 67.9 | Q8NFJ9 | 75.71 | 65.1 |
| *BBS10* | Bardet-Biedl syndrome 10 | *bbs10* | ENSDARG00000069515 | 30.3 | Q8TAM1 | 61.63 | 43.78 |
| *BBS2* | Bardet-Biedl syndrome 2 | *bbs2* | ENSDARG00000041621 | 73.9 | Q9BXC9 | 398.21 | 459.19 |
| *ARL6* | ADP-ribosylation factor-like 6 | *arl6* | ENSDARG00000032056 | 88.2 | Q9H0F7 | 145.41 | 363.94 |
| *BBS4* | Bardet-Biedl syndrome 4 | *bbs4* | ENSDARG00000063522 | 74.6 | Q96RK4 | 64.55 | 93.27 |
| *BBS5* | Bardet-Biedl syndrome 5 | *bbs5* | ENSDARG00000039827 | 90.9 | Q8N3I7 | 62.86 | 86.37 |
| *MKKS* | McKusick-Kaufman syndrome | *mkks* | ENSDARG00000016139 | 41.4 | Q9NPJ1 | 117.59 | 61.77 |
| *BBS7* | Bardet-Biedl syndrome 7 | *bbs7* | ENSDARG00000059911 | 76.2 | Q8IWZ6 | 264.49 | 250 |
| *TTC8* | Tetratricopeptide repeat domain 8 | *ttc8* | ENSDARG00000099481 | 72.1 | Q8TAM2 | 54.44 | 72.45 |
| *BBS9* | Bardet-Biedl syndrome 9 | *bbs9* | ENSDARG00000079217 | 63.7 | Q3SYG4 | 27.88 | 39.11 |
| *TRIM32* | Tripartite motif containing 32 | *trim32* | ENSDARG00000102505 | 63.6 | Q13049 | 231.96 | 166.05 |
| *BBS12* | Bardet-Biedl syndrome 12 | *bbs12* | ENSDARG00000016112 | 30.7 | Q6ZW61 | 107.77 | 78.67 |
| *MSK1* | Ribosomal protein S6 kinase, polypeptide 5 | *rps6ka5* | ENSDARG00000060551 | 76.3 | O75582 | 214.56 | 159.13 |
| *CEP290* | Centrosomal protein 290 | *cep290* | ENSDARG00000062727 | 57.8 | O15078 | 1022.8 | 746.67 |
| *WDPCP* | WD repeat containing planar cell polarity effector | *wdpcp* | ENSDARG00000074590 | 44 | O95876 | 193.09 | 313.44 |
| *SDCCAG8* | Serologically defined colon cancer antigen 8 | *sdccag8* | ENSDARG00000078947 | 44.1 | Q86SQ7 | 292.54 | 276.25 |
| *LZTFL1* | Leucine zipper transcription factor-like 1 | *lztfl1* | ENSDARG00000105010 | 69.9 | Q9NQ48 | 587.57 | 863.58 |
| *BBIP1* | BBSome interacting protein 1 | *bbip1* | ENSDARG00000071046 | 76.9 | A8MTZ0 | 529.88 | 759.02 |
| *IFT27* | Intraflagellar transport 27 homolog | *ift27* | ENSDARG00000099279 | 55.6 | Q9BW83 | 371.17 | 326.64 |
| *IFT172* | Intraflagellar transport 172 | *ift172* | ENSDARG00000041870 | 75.1 | Q9UG01 | 699.57 | 760.76 |
| *C8orf37* | Cilia and flagella associated protein 418 | *cfap418* | ZDB-GENE-100921-86 | 45.5 | Q96NL8 | 0 | 0 |
| *NPHP1* | Nephronophthisis 1 | *nphp1* | ENSDARG00000009046 | 44.3 | O15259 | 68.04 | 113.84 |
| *IFT74* | Intraflagellar transport 74 | *ift74* | ENSDARG00000023495 | 59.6 | Q96LB3 | 1039.98 | 1486.45 |
| *SCAPER* | S-phase cyclin A-associated protein in the ER | *scaper* | ENSDARG00000023104 | 74.6 | Q9BY12 | 470.19 | 458.18 |
| **SLS** |  |  |  |  |  |  |  |
| *IQCB1* | IQ motif containing B1 | *iqcb1* | ENSDARG00000093431 | 36.8 | Q15051 | 6.289 | 16.05 |
| *INVS* | Inversin | *invs* | ENSDARG00000002213 | 54.2 | Q9Y283 | 332.77 | 105.35 |
| *NPHP3* | Nephronophthisis 3 | *nphp3* | ENSDARG00000078261 | 69.1 | Q7Z494 | 294.91 | 135.9 |
| *NPHP4* | Nephronophthisis 4 | *nphp4* | ENSDARG00000069014 | 45.8 | O75161 | 156.75 | 161.49 |
| *CEP164* | Centrosomal Protein 164 | *cep164* | ZDB-GENE-120821-1 | 74 | Q9UPV0 | 0 | 0 |
| **JBTS** |  |  |  |  |  |  |  |
| *AHI1* | Abelson helper integration site 1 | *ahi1* | ENSDARG00000044056 | 43 | Q8N157 | 314.93 | 457.9 |
| *INPP5E* | Inositol polyphosphate-5-phosphatase E | *inpp5e* | ENSDARG00000103926 | 56.3 | Q9NRR6 | 210.92 | 280.01 |
| *TMEM67* | Transmembrane protein 67 | *tmem67* | ENSDARG00000076752 | 59.3 | Q5HYA8 | 139.89 | 219.73 |
| *RPGRIP1L* | RPGRIP1 like | *rpgrip1l* | ENSDARG00000051754 | 48.9 | Q68CZ1 | 384.1 | 299.87 |
| *CC2D2A* | Coiled-coil and C2 domain containing 2A | *cc2d2a* | ENSDARG00000090971 | 57.4 | Q9P2K1 | 926.46 | 731.35 |
| *ARL13B* | ADP ribosylation factor like GTPase 13B | *arl13b* | ENSDARG00000012763 | 61.9 | Q3SXY8 | 372.87 | 543.83 |
| *ARL3* | ADP ribosylation factor like GTPase 3 | *arl3a arl3b* | ENSDARG00000056794 ENSDARG00000071409 | 95.1 94.5 | P36405 | 57.81 322.28 | 45.44 344.94 |
| *CPLANE1* | Ciliogenesis and Planar Polarity Effector 1 | *cplane1* | ZDB-GENE-091204-92 | 36 | Q9H799 | 0 | 0 |
| *CEP104* | Centrosomal protein 104 | *cep104* | ENSDARG00000060361 | 52.4 | O60308 | 157.08 | 247.06 |
| *TMEM237* | Transmembrane protein 237 | *tmem237a tmem237b* | ZDB-GENE-070402-3 ZDB-GENE-040912-63 | 52.6 52.3 | Q96Q45 | 10.70 255.91 | 0 137.77 |
| *ARMC9* | Armadillo repeat containing 9 | *armc9* | ENSDARG00000087299 | 58.7 | Q7Z3E5 | 1556.28 | 7046.43 |
| *CEP120* | Centrosomal protein 120 | *cep120* | ENSDARG00000091326 | 58.8 | Q8N960 | 390.32 | 192.42 |
| *CEP41* | Centrosomal protein 41 | *cep41* | ENSDARG00000038500 | 57.1 | Q9BYV8 | 44.03 | 41.5 |
| *CSPP1* | Centrosome and spindle pole associated protein 1a | *cspp1a cspp1b* | ENSDARG00000100236 ENSDARG00000091628 | 38.6 32.2 | A0A7I2V5P5 | 158.88 495.48 | 181.33 395.43 |
| *SUFU* | SUFU negative regulator of hedgehog signaling | *sufu* | ENSDARG00000056801 | 81.3 | Q9UMX1 | 968.32 | 814.65 |
| *TMEM138* | Transmembrane protein 138 | *tmem138* | ENSDARG00000090543 | 67.9 | Q9NPI0 | 228.26 | 309.4 |
| *TMEM216 (CORS2)* | Transmembrane protein 216 | *tmem216* | ENSDARG00000091576 | 59 | Q9P0N5 | 88.33 | 36.56 |
| *POC1B* | POC1 centriolar protein B | *poc1b* | ENSDARG00000021110 | 56 | Q8TC44 | 417.6 | 5593.25 |
| *TCTN1* | Tectonic family member 1 | *tctn1* | ENSDARG00000078447 | 40.3 | Q2MV58 | 157.04 | 113.65 |
| *TCTN2* | Tectonic family member 2 | *tctn2* | ENSDARG00000035633 | 37.4 | Q96GX1 | 195.71 | 145.06 |
| *PIBF1* | Progesterone immunomodulatory binding factor 1 | *pibf1* | ENSDARG00000013006 | 65.6 | Q8WXW3 | 346.17 | 140.42 |
| *KIAA0586* | Talpid3 | *talpid3* | ENSDARG00000097772 | 32.1 | Q9BVV6 | 234.87 | 156.42 |
| *KIF7* | Kinesin Family Member 7 | *kif7* | ENSDARG00000033099 | 57.1 | Q2M1P5 | 880.23 | 385.34 |
| *KATNIP* | Katenin Interacting Protein | *katnip* | ZDB-GENE-091204-173 | 48.9 | O60303 | 0 | 0 |
| *ZNF423* | Zinc Finger Protein 423 | *znf423* | ENSDARG00000095732 | 76.8 | Q2M1K9 | 480.52 | 538.35 |
| *TTC21B* | Tetratricopeptide Repeat Domain 21B | *ttc21b* | ENSDARG00000012368 | 68.7 | Q7Z4L5 | 358.62 | 266.29 |
| **MKS** |  |  |  |  |  |  |  |
| *TMEM231* | Transmembrane Protein 231 | *tmem231* | ENSDARG00000042272 | 57.9 | Q9H6L2 | 171.75 | 110.67 |
| *B9D1* | B9 protein domain 1 | *b9d1* | ENSDARG00000011727 | 83.2 | Q9UPM9 | 171.95 | 156.2 |
| *MKS1* | MKS transition zone complex subunit 1 | *mks1* | ENSDARG00000059657 | 60.4 | Q9NXB0 | 214.56 | 159.13 |
| *B9D2* | B9 domain containing 2 | *b9d2* | ENSDARG00000017385 | 75.9 | Q9BPU9 | 127.67 | 121.14 |
| *OFD1* | OFD1 centriole and centriolar satellite protein | *ofd1* | ENSDARG00000000529 | 34.5 | O75665 | 405.27 | 322.68 |
| *WDR19* | WD repeat domain 19 | *wdr19* | ENSDARG00000037406 | 73.6 | Q8NEZ3 | 373.68 | 176.98 |
| *KIF14* | Kinesin family member 14 | *kif14* | ENSDARG00000062187 | 55.3 | Q15058 | 256.21 | 113.68 |
| *CEP55* | Centrosomal Protein 55 | *cep55l* | ENSDARG00000102349 | 37.1 | Q53EZ4 | 522.15 | 163.15 |
| *TXNDC15* | Thioredoxin domain containing 15 | *txndc15* | ENSDARG00000110357 | 62.2 | Q96J42 | 316.08 | 374.82 |
| **NPHP** |  |  |  |  |  |  |  |
| *GLIS2* | GLIS family zinc finger 2 | *glis2a glis2b* | ENSDARG00000078388 | 50.5 52.6 | Q9BZE0 | 399.28 21.85 | 91.16 12.197 |
| *NEK8* | NIMA-related kinase 8 | *nek8* | ENSDARG00000045626 | 75 | Q86SG6 | 73.19 | 112.19 |
| *ANKS6* | Ankyrin Repeat and Sterile Alpha Motif DomainContaining 6 | *anks6* | ZDB-GENE-041010-213 | 58.7 | Q68DC2 | 0 | 0 |
| *CEP83* | Centrosomal protein 83 | *cep83* | ENSDARG00000101236 | 55.4 | Q9Y592 | 527.75 | 394.88 |
| *DCDC2* | Doublecortin domain containing 2 | *dcdc2b* | ENSDARG00000053744 | 41.2 | Q9UHG0 | 156.19 | 218.75 |
| *MAPKBP1* | Mitogen-activated protein kinase binding protein 1 | *mapkbp1* | ENSDARG00000103746 | 49.2 | O60336 | 194.13 | 146.84 |
| *XPNPEP3* | X-prolyl aminopeptidase 3, mitochondrial | *xpnpep3* | ENSDARG00000007916 | 57.8 | Q9NQH7 | 471.08 | 1003.63 |
| *ATXN10* | Ataxin 10 | *atxn10* | ENSDARG00000008439 | 39.3 | Q9UBB4 | 175.35 | 183.32 |
| *SLC41A1* | Solute Carrier Family 41 Member 1 | *slc41a1* | ENSDARG00000070214 | 80.3 | SLC41A1 | 18.75 | 42.92 |

Supplementary Table 2: Zebrafish cystic kidney disease genes have different expression level in kidney

| **Zebrafish Homolog** | **Kidney RPKM (M)** | **Kidney RPKM (F)** |
| --- | --- | --- |
| **Group 1** | | |
| *armc9* | 7046.43 | 1556.28 |
| *poc1b* | 5593.25 | 417.6 |
| *ganabb* | 3039.12 | 3146.28 |
| *dnajb11* | 1772.92 | 2266.67 |
| *ift74* | 1486.45 | 1039.98 |
| *ganaba* | 1300.4 | 1035.1 |
| *xpnpep3* | 1003.63 | 471.08 |
|  | **Group 2** |  |
| *lztfl1* | 863.58 | 587.57 |
| *sufu* | 814.65 | 968.32 |
| *ift172* | 760.76 | 699.57 |
| *bbip1* | 759.02 | 529.88 |
| *cep290* | 746.67 | 1022.8 |
| *cc2d2a* | 731.35 | 926.46 |
| *arl13b* | 543.83 | 372.87 |
| *znf423* | 538.35 | 480.52 |
| *bbs2* | 459.19 | 398.21 |
| *scaper* | 458.18 | 470.19 |
| *ahi1* | 457.9 | 314.93 |
| *cspp1b* | 395.43 | 495.48 |
| *cep83* | 394.88 | 527.75 |
| *kif7* | 385.34 | 880.23 |
| *alg9* | 376.21 | 513.43 |
| *txndc15* | 374.82 | 316.08 |
| *arl6* | 363.94 | 145.41 |
| *arl3b* | 344.94 | 322.28 |
| *ift27* | 326.64 | 371.17 |
| *dzip1l* | 325.15 | 158.85 |
| *ofd1* | 322.68 | 405.27 |
| *wdpcp* | 313.44 | 193.09 |
| *tmem138* | 309.4 | 228.26 |
| *rpgrip1l* | 299.87 | 384.1 |
| *inpp5e* | 280.01 | 210.92 |
| *sdccag8* | 276.25 | 292.54 |
| *ttc21b* | 266.29 | 358.62 |
| *bbs7* | 250 | 264.49 |
| *cep104* | 247.06 | 157.08 |
| *tmem67* | 219.73 | 139.89 |
| *dcdc2b* | 218.75 | 156.19 |
| *cep120* | 192.42 | 390.32 |
| *ift140* | 189.76 | 393.42 |
| *atxn10* | 183.32 | 175.35 |
| *cspp1a* | 181.33 | 158.88 |
| *wdr19* | 176.98 | 373.68 |
| *trim32* | 166.05 | 231.96 |
| *cep55l* | 163.15 | 522.15 |
| *nphp4* | 161.49 | 156.75 |
| *rps6ka5* | 159.13 | 214.56 |
| *mks1* | 159.13 | 214.56 |
| *kiaa0586* | 156.42 | 234.87 |
| *b9d1* | 156.2 | 171.95 |
| *mapkbp1* | 146.84 | 194.13 |
| *tctn2* | 145.06 | 195.71 |
| *pibf1* | 140.42 | 346.17 |
| *tmem237b* | 137.77 | 255.91 |
| *nphp3* | 135.9 | 294.91 |
| *b9d2* | 121.14 | 127.67 |
| *nphp1* | 113.84 | 68.04 |
| *kif14* | 113.68 | 256.21 |
| *tctn1* | 113.65 | 157.04 |
| *pkd2* | 112.29 | 71.28 |
| *nek8* | 112.19 | 73.19 |
| *tmem231* | 110.67 | 171.75 |
| *invs* | 105.35 | 332.77 |
|  | **Group 3** |  |
| *bbs4* | 93.27 | 64.55 |
| *glis2a* | 91.16 | 399.28 |
| *bbs5* | 86.37 | 62.86 |
| *bbs12* | 78.67 | 107.77 |
| *ttc8* | 72.45 | 54.44 |
| *bbs1* | 65.1 | 75.71 |
| *mkks* | 61.77 | 117.59 |
| *pkd1a* | 47.19 | 61.99 |
| *arl3a* | 45.44 | 57.81 |
| *bbs10* | 43.78 | 61.63 |
| *slc41a1* | 42.92 | 18.75 |
| *cep41* | 41.5 | 44.03 |
| *bbs9* | 39.11 | 27.88 |
| *tmem216* | 36.56 | 88.33 |
| *iqcb1* | 16.05 | 6.289 |
| *glis2b* | 12.197 | 21.85 |
| *pkd1b* | 3.29 | 5.34 |
|  | **Group 4** |  |
| *cfap418* | 0 | 0 |
| *cep164* | 0 | 0 |
| *cplane1* | 0 | 0 |
| *tmem 237a* | 0 | 10.7 |
| *katnip* | 0 | 0 |
| *anks6* | 0 | 0 |

Supplementary Table 3: qPCR Primer Sequences

| **Gene Name** | **Primer Sequence** |
| --- | --- |
| *tnfrsf9a-*F | 5’ AAATGCTGGCTGGTTTTCC |
| *tnfrsf9a-*R | 5’ TGCCAGGTTCACATGGTTTA |
| *ccnb3-*F | 5’ TTGCAAAGATCCGATTCCTC |
| *ccnb3-*R | 5’ TCCACCAGAATCGCTCTCAT |
| *nfkbiaa-*F | 5’ TCAAACAGTGCCAGAACGAC |
| *nfkbiaa-*R | 5’ CAGGTGCTGGGTCTGAATCT |
| *casp3b-*F | 5’ TTCAGATGGATGCCAAATCA |
| *casp3b-*R | 5’ TCATCTGTGAGACGGTCAGG |
| *il1b-*F | 5’ CGCTTGCAATGAGCTACAGA |
| *il1b-*R | 5’ CACTTCACGCTCTTGGATGA |
| *agxtb-*F | 5’ GGATTCAGTGGCATCATTGG |
| *agxtb-*R | 5’ CTGGTTTGCCATCATTACCC |
| *ahr1a-*F | 5’ GTTCCAGCCAACAAGCAAAT |
| *ahr1a-*R | 5’ TCATGCCGGTCTTCAGTATG |
| *hsp90aa1.2-*F | 5’ AGCAGCAGATGATGGAGGAT |
| *hsp90aa1.2-*R | 5’ CGCTCTTCTTTGTTGGGAAT |
| *cbsb-*F | 5’ GTGGCAGTGTGAAGGACAGA |
| *cbsb-*R | 5’ AATCTCAGCACCCAGAGCAC |
| *ahcyl2a-*F | 5’ CTCAGAATGAAGTGGCAGCA |
| *ahcyl2a-*R | 5’ TGTGAATGCCTGTGATGCTT |


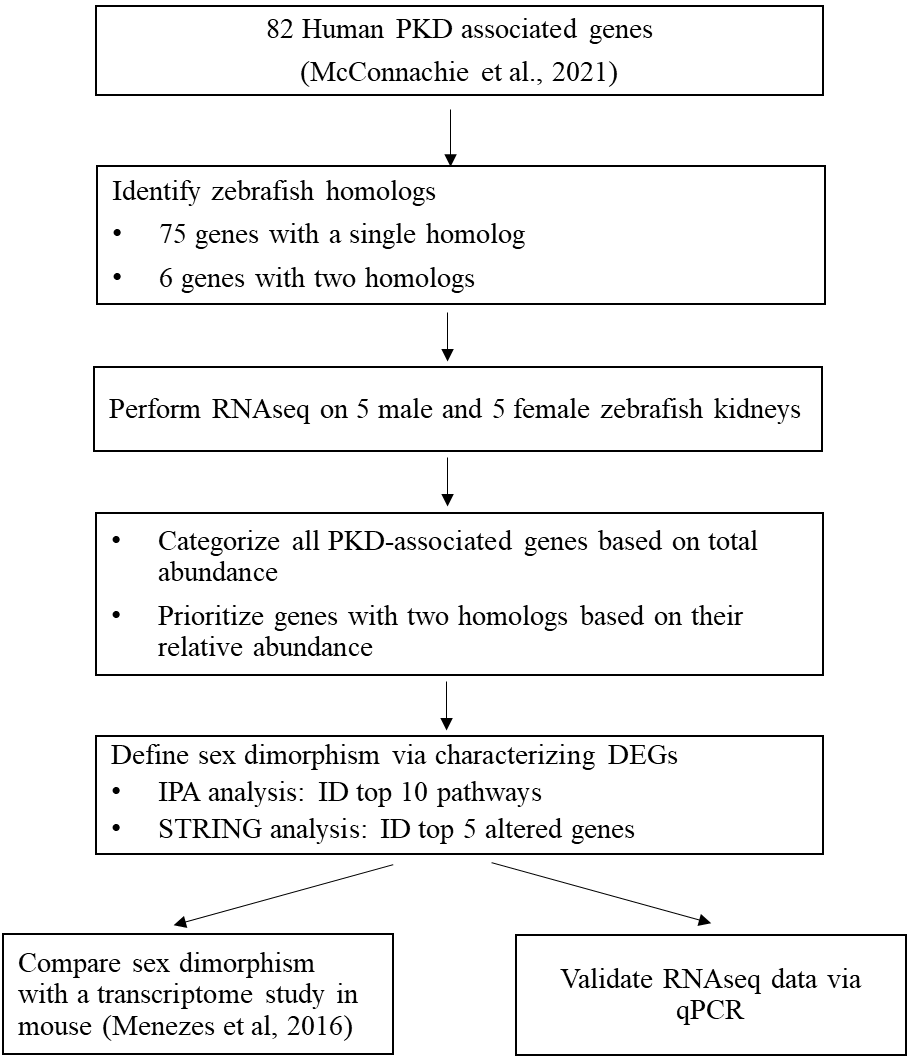


Supplementary Figure 1. Flowchart on experimental design


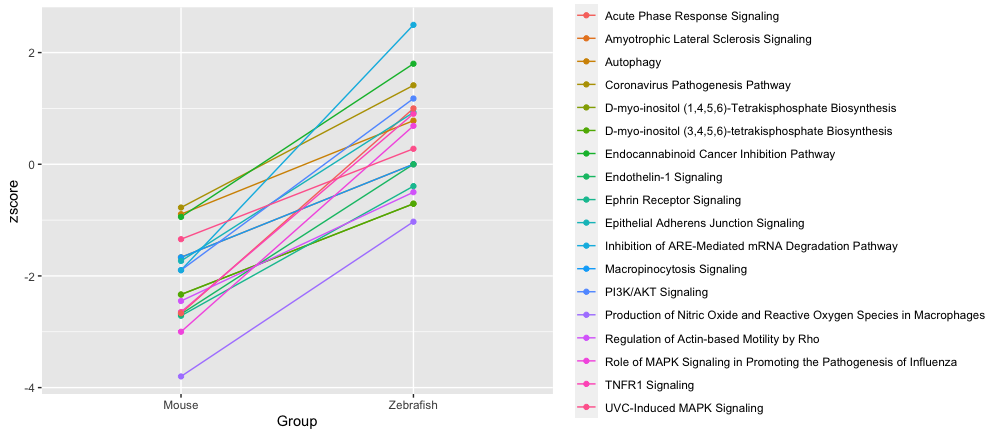

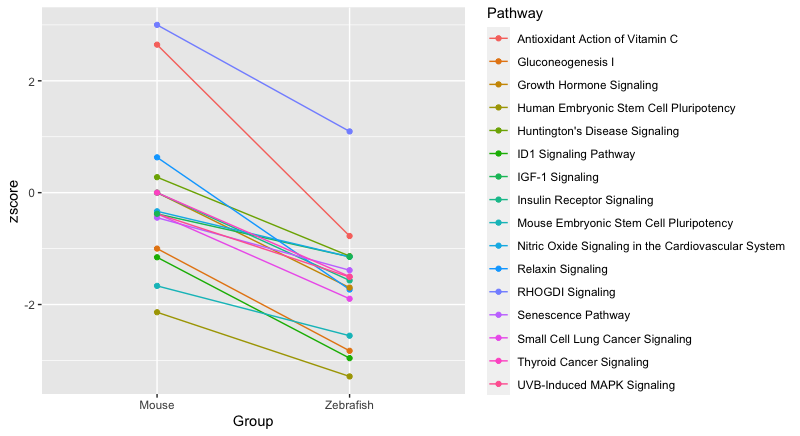


**A**

**B**

Supplementary Figure 2. 26 pathways have an absolute z-score difference >2.5. (**A**) Z-scores of pathways that are higher in zebrafish vs mouse. (**B**) Z-scores of pathways that are lower in zebrafish vs mouse.


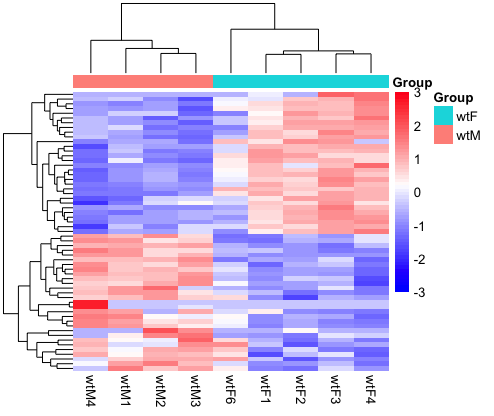

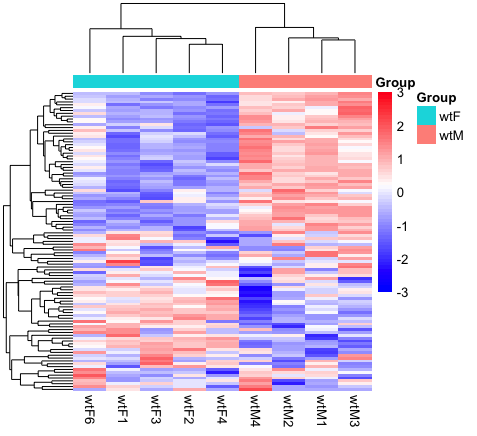

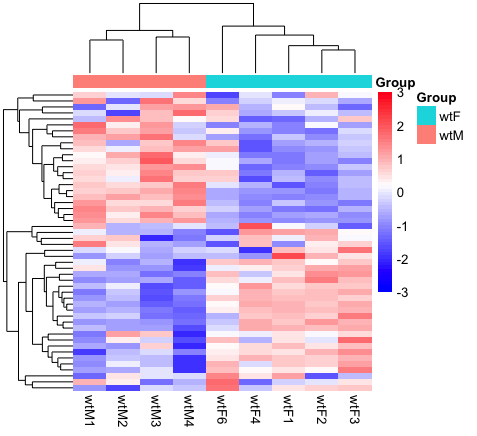

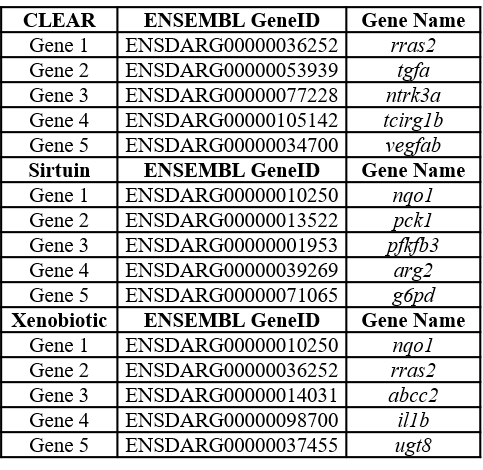


Supplementary Figure 3. Differentially expressed genes in three representative signaling pathways underlying sex dimorphism in zebrafish kidneys. (**A**) Heatmap of CLEAR genes in male vs female zebrafish. (**B**) Heatmap of Sirtuin genes in male vs female zebrafish. (**C**) Heatmap of Xenobiotic genes in male vs female zebrafish. (**D**) Table of top 5 DEGs in CLEAR, Sirtuin, and Xenobiotic pathways.

**A**

**B**

**C**

**D**
